# Left and Right Ventricular Impairment Shortly After Correction of Tetralogy of Fallot

**DOI:** 10.1007/s00246-020-02355-6

**Published:** 2020-05-04

**Authors:** Covadonga Terol, Vivian P. Kamphuis, Mark G. Hazekamp, Nico A. Blom, Arend D. J. Ten Harkel

**Affiliations:** 1 Division of Paediatric Cardiology, Department of Paediatrics, Leiden University Medical Centre, Albinusdreef 2, 2333 ZA Leiden, The Netherlands; 2 Netherlands Heart Institute, Utrecht, The Netherlands; 3 Department of Cardiothoracic Surgery, Leiden University Medical Center, Leiden, The Netherlands; 4 Division of Paediatric Cardiology, Department of Paediatrics, Academic Medical Centre, Amsterdam, The Netherlands

**Keywords:** Tetralogy of fallot, Strain, Tissue and strain doppler echocardiography, Cardiac surgery, Color doppler tissue imaging, Doppler tissue imaging

## Abstract

Surgical repair of Tetralogy of Fallot (ToF) is usually performed in the first months of life with low early postoperative mortality. During long-term follow-up, however, both right (RV) and left ventricular (LV) performances may deteriorate. Tissue Doppler imaging (TDI) and speckle tracking echocardiography (ST) can unmask a diminished RV and LV performance. The objective of the current study was to assess the cardiac performance before and shortly after corrective surgery in ToF patients using conventional, TDI and ST echocardiographic techniques. Thirty-six ToF patients after surgery were included. Transthoracic echocardiography including TDI and ST techniques was performed preoperatively and at hospital discharge after surgery (10 days to 4 weeks after surgery). Median age at surgery was 7.5 months [5.5–10.9]. Regarding the LV systolic function there was a significant decrease in interventricular septum (IVS) *S*′ at discharge as compared to preoperatively (pre IVS *S*′ = 5.4 ± 1.4; post IVS *S*′ = 3.9 ± 1.2; *p* < 0.001) and in global longitudinal peak strain (GLS) (pre = − 18.3 ± 3.4; post = − 14.2 ± 4.1; *p* = 0.003); but not in the fractional shortening (FS). Both conventional and TDI parameters showed a decrease in diastolic function at discharge. Tricuspid Annular Plane Systolic Excursion and RV *S*′ were significantly lower before discharge. When assessing the RV diastolic performance, only the TDI demonstrated a RV impairment. There was a negative correlation between age at surgery and postoperative LV GLS (*R* = − 0.41, *p* = 0.031). There seems to be an impairment in left and right ventricle performance at discharge after ToF corrective surgery compared to preoperatively. This is better determined with TDI and ST strain imaging than with conventional echocardiography.

## Introduction

With a frequency of one in 3.500 births, Tetralogy of Fallot (ToF) is one of the most frequent congenital heart defects (CHD). At the present time, complete surgical repair is usually performed in the first months of life with low early postoperative mortality (1.1%) [1, 2], and most patients survive into adulthood with a 25-year survival of around 95%. However, long-term morbidity is significant and mainly the result of chronic pulmonary regurgitation due to the relief of right ventricular outflow tract obstruction during surgical repair. The effects of chronic RV volume overload on RV size and RV function become evident in early adulthood with clinical heart failure, atrial and ventricular arrhythmias, and sudden death. In addition, around 20% of adults with repaired ToF eventually develop left ventricular (LV) dysfunction [3]. Preoperative RV hypertrophy is usually present, but it is as yet unclear if RV function has been normalized at the time of discharge postoperatively.

Echocardiography is the standard in clinical follow-up of ToF patients. Previous studies have shown that tissue Doppler imaging (TDI) and speckle tracking echocardiography (ST) can unmask a diminished right (RV) and left ventricular (LV) performance after correction of various CHD [4–11]. TDI permits the direct quantification of myocardial velocities [12] with a relative geometric independence but is angle-dependent and only enables the analysis in the longitudinal direction [6]. ST is not angle-dependent, is not based on geometrical assumption and allows multidirectional assessment of ventricular function, making possible to study both regional and global function in both ventricles [13]. Impaired biventricular performance on echocardiography directly after cardiac surgical correction has been shown in CHD patients after VSD closure, coarctation repair and arterial switch operation [4, 5, 14, 15]. The objective of the current study was to assess the cardiac performance before and shortly after corrective surgery in ToF patients using conventional, TDI and ST echocardiographic techniques.

## Methods

### Study Subjects

This study was a prospective study and included ToF patients who underwent surgical correction at the Leiden University Medical Center. Institutional medical ethical approval was obtained and written informed consent was obtained from all participants and/or parents/guardians as appropriate. Clinical data such as presence of right bundle brunch block (RBBB) on the electrocardiogram or duration of postoperative pleural effusion, and surgical data (type of RV outflow tract relief and cardiopulmonary bypass time) were obtained from the electronic medical records. Corrective surgery consisted of patch closure of the ventricular septal defect and relief of the RVOT obstruction requiring a transannular patch in 69% (25/36) of the patients. Transthoracic echocardiography including TDI and ST techniques was performed preoperatively and at hospital discharge after surgery (10 days to 4 weeks after surgery) to establish a short-term follow-up.

### Echocardiography

Echocardiography was performed using a commercially available system (Vivid-7.0.0, General Electric Vingmed Ultrasound, Horten, Norway). The images were stored in digital format to allow off-line analyses using EchoPac version 11.1.8 (General Electric Vingmed). All recordings were performed while the patient was in sinus rhythm and without any sedation. Spectral Doppler, TDI tracings and ST strain analyses were performed from the apical four-chamber (A4C) view. Additionally, M-mode recordings of the LV long axis were recorded. Each parameter was measured in three consecutive beats and averaged. Echocardiographic analysis was performed by one observer (CT) and reviewed by a second observer (ADJH).

#### Conventional Echocardiography

LV systolic performance was assessed in M-mode recordings of the LV long axis using LV FS in %. To calculate FS, LV internal diameter at end-diastole (LVIDd) and LV internal diameter at end-systole (LVIDs) were assessed (in mm) and FS was calculated as follows: ((LVIDd−LVIDs)/LVIDd) × 100%. LV diastolic performance was assessed by measurements of peak left ventricular early-wave velocity (*E*) (in m/s) and peak atrial contraction wave velocity (*A*) (in m/s) from spectral Doppler tracings recorded in the A4C at the tip of the mitral leaflets. Additionally, the *E*/*A* ratio was calculated. RV systolic performance was assessed using TAPSE (in mm) measurements in two-dimensional M-mode recordings of the A4C view as previously described [16]. Finally, RV diastolic performance was assessed using *E* and *A* measurements and *E*/*A* ratio assessed from spectral Doppler tracings recorded at the tip of the tricuspid valve.

#### Tissue Doppler Imaging

The TDI images were recorded from the A4C view during three consecutive cardiac cycles and all values were averaged. The angle of insonation was adjusted to align the ultrasound beam along the direction of myocardial motion. The LV lateral wall, the interventricular septum (IVS) and the RV free wall longitudinal myocardial velocity curves were obtained by placing the cursor at the basal part of each region. Subsequently, peak systolic velocities (*S*′) and peak early (*E*′) and late diastolic velocities (*A*′) were assessed in each myocardial velocity curve. In addition, *E*/*E*′, a diastolic parameter strongly correlated with ventricular filling pressure [17] was calculated.

#### Speckle Tracking

LV systolic performance was also evaluated using ST strain analyses performed in grayscale images of the A4C view (longitudinal analysis). Images were obtained with optimized sector width and frame rate (preferably 60–90 frames/second). In these images manual endocardial border tracing at end-systole was used to set the region of interest. The region of interest was automatically divided into 6 segments (basal septal (BS), mid septal (MS), apical septal (AS), basal lateral (BL) mid lateral (ML) and apical lateral (AL)). In each segment, tracking quality was automatically evaluated and this resulted in automatic rejection or acceptation of the segment. Although the observer could override this automatic decision based on visual evaluation [18, 19], this was used very conservatively and regarded as feasible when at least five segments were scored as adequate. Data obtained by ST were displayed in longitudinal time-strain curves for each segment [20]. From these time-strain curves segmental peak strain was obtained. Peak strain (PS) was defined as the most negative strain value at any time point during one cardiac cycle. Finally, global longitudinal peak strain (GLS) defined as the average of the individual segmental curves was obtained [19].

### Statistical Analysis

Data analysis was performed using SPSS Statistics software (v. 23.0 IBM SPSS, Chicago, IL). Variables were tested for normal distribution using the Shapiro–Wilk test. Continuous data were expressed as mean ± standard deviation (SD) or as median [inter-quartile range] where suitable. The paired samples t test or, in case of non-normality the Wilcoxon signed-rank test, was used to assess differences between echocardiographic parameters, including TDI and ST measurements, preoperatively and at discharge. Correlations between the postoperative echocardiographic parameters and the clinical parameters were calculated as Pearson or Spearman correlation coefficient depending on data distribution. Intraobserver and interobserver variability were assessed in randomly selected 15 subjects. Intraobserver variability was determined by having 1 observer remeasurement after 6 months. Interobserver variability was determined by a second observer who was blinded to the clinical and the STE findings. Interobserver and intraobserver reproducibility were evaluated by means of intraclass correlation coefficient (ICC). *p *values < 0.05 were accepted as statistically significant.

## Results

In the current study, 36 ToF patients were included. Table 1 shows the patient characteristics of the study group. In 6 patients, a shunt was necessary because of cyanotic spells, in two of them despite the use of propranolol. Complete surgical repair was performed at a median age of 7.5 months [5.5–10.9], 7 patients were > 12 months. Preoperative saturation was 90% [85–94]. Right Ventricular Outflow Tract (RVOT) reconstruction was performed with the use of a transannular patch in 25 patients (69%), and only infundibulectomy and/or pulmonary valve commissurotomy in 11 patients (31%). Cardiopulmonary bypass time was 84 min [75–98]. Postoperatively there was a median of 1 day [IQR: 1–4 days] of pleural effusion. A RBBB was present in 74% of the patients. At discharge, all patients used diuretics.

**Table 1:** Patient characteristics

	*N* = 36
Males (%)	21 (58)
Age (months)	7.5 [5.5–10.9]
Weight (kg)	7.4 ± 1.2
Height (cm)	69.3 [66.8–71.3]
BMI (m^2^)	15 ± 1.8
Preoperative SatO_2_ (%)	90 [85–94]
Previous shunt (number; %)	6 (16.7)
Transannular patch (number; %)	25 (69)
Cardiopulmonary bypass time (mins)	84 [75–98]
Pleural effusion after surgery (days)	1 [1–4]

Data shown as number (%), mean ± SD or median [IQR]

### Left Ventricular Performance

**Fig. 1:**
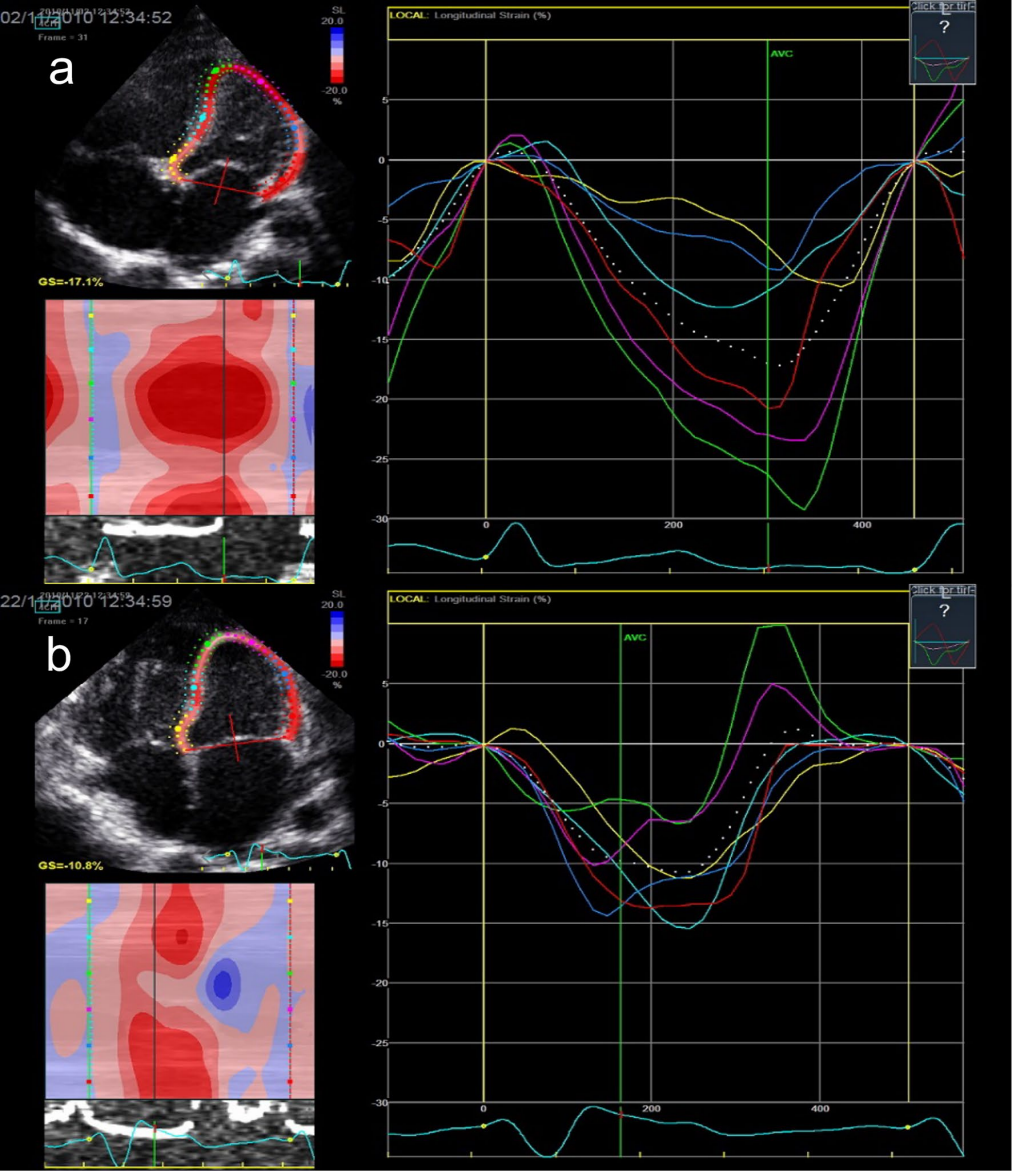
Longitudinal strain of a male patient with Tetralogy of Fallot: **a** before surgery (GLS − 17.1%) and **b** before discharge after surgery (GLS − 10.8%). *GLS* global longitudinal peak strain

Table 2 presents the conventional and TDI echocardiographic LV systolic and diastolic performance parameters for the patients before surgery and at discharge. Change in systolic function was demonstrated by TDI, with a significant decrease in IVS *S*′ at discharge as compared to preoperatively, but no differences were found in the FS. Systolic deterioration was also assessed by GLS being significantly lower at discharge compared to the values before surgery (Table 3). Figure 1 sows the longitudinal strain of a male patient before surgery and after discharge. Both conventional and TDI parameters showed a decrease in diastolic function at discharge (increase in spectral left ventricular Doppler E/A ratio and decrease in IVS *E*′ and IVS *A*′).

**Table 2:** Left ventricular functional echocardiographic measurements

	Preoperatively	Before discharge	Difference (post–pre)	*p *value
	Mean ± SD or Median [IQR]	Mean ± SD or Median [IQR]	Mean ± SD or Median [IQR]	
Systolic				
FS (%)	37.1 ± 7.1	34.4 ± 6.7	− 2.5 ± 7.1	0.14
TDI LV				
Lateral wall				
* S*′ (cm/s)	5.2 ± 1.4	5.8 ± 1.6	0.6 ± 2.1	0.12
IVS				
* S*′ (cm/s)	5.3 [4.0–6.7]	3.9 ± 1.2	− 1.4 ± 1.8	< 0.001*
Diastolic				
MV Doppler flow				
E (m/s)	1.0 ± 0.3	1.0 [0.8–1.2]	0.04 ± 0.4	0.42
* A* (m/s)	0.8 ± 0.2	0.8 ± 0.2	− 0.1 ± 0.3	0.06
* E*/*A*	1.1 [1.0–1.2]	1.4 ± 0.5	0.3 ± 0.5	0.006*
TDI LV				
Lateral wall				
* E*′ (cm/s)	9.8 ± 3.4	9.7 ± 2.7	− 0.006 ± 4.1	0.99
* A*′ (cm/s)	5.9 ± 1.8	6.0 ± 2.3	0.4 ± 3.1	0.50
* E*/*E*′	9.5 [8.2–12.3]	10.2 [8.7–12.3]	0.4 ± 4.9	0.59
IVS				
* E*′ (cm/s)	8.9 ± 2.7	7.6 ± 2.5	− 1.4 ± 3.3	0.03*
* A*′ (cm/s)	7.6 ± 2.8	5.0 [3.2–7.8]	− 2.6 ± 2.8	0.001*

*Means significant. *p *value determined with paired samples *t* test or Wilcoxon signed-rank test

*FS* fractional shortening, *TDI* tissue doppler image, *LV* left ventricle, *MV* mitral valve, *IVS* interventricular septum

**Table 3:** Strain analysis in longitudinal 6 segments model

	Preoperatively	Before discharge	Difference (post–pre)	*p* value
	Mean ± SD or Median [IQR]	Mean ± SD or Median [IQR]	Mean ± SD or Median [IQR]	
Global longitudinal peak strain (%)				
	− 18.3 ± 3.4	− 14.2 ± 4.1	3.4 ± 5.1	0.003*
Time to global peak strain (msec)				
	288.9 ± 29.8	267.2 ± 33.8	− 20.7 ± 47.1	0.04*
Regional peak strain (%)				
BS	− 16.4 ± 3.9	− 12.8 ± 4.2	− 3.2 ± 5.9	0.012*
MS	− 17.3 ± 3.3	− 15.5 ± 3.4	− 1.9 ± 4.7	0.065
AS	− 25.2 [− 28.8 to − 22.1]	− 9.9 ± 8.1	− 16.9 ± 11.7	< 0.001*
AL	− 17.2 [24–14.7]	− 10.2 [16.8–7.1]	− 5.7 ± 12.9	0.07
ML	− 12.6 ± 4.8	− 10 [16.4–8.4]	− 0.5 ± 8.2	0.903
BL	− 25.9 ± 5.8	− 23.4 ± 7	− 1.7 ± 10.4	0.439

*Means significant. *p *value determined with paired samples *t* test or Wilcoxon signed-rank test

*BS* basal septal, *MS* mitral septal, *AS* apical septal, *AL* apical lateral, *ML* mid lateral, *BL* basal lateral

### Right Ventricular Performance

Table 4 presents the conventional and TDI echocardiography RV systolic and diastolic performance parameters for the patients before the surgery and at discharge. TAPSE and RV *S*′ were significantly lower before discharge as compared to preoperative values. When assessing the RV diastolic performance, RV *E*′ and *A*′ were significantly decreased and *E*/*E*′ was significantly increased. In contrast, the tricuspid spectral Doppler parameters did not show any differences.

**Table 4:** Right ventricular functional echocardiographic measurements

	Preoperatively	Before discharge	Difference (post–pre)	*p *value
	Mean ± SD or Median [IQR]	Mean ± SD or Median [IQR]	Mean ± SD or Median [IQR]	
Systolic				
TAPSE	12.9 ± 2.6	6.5 ± 1.6	− 6.5 ± 3.1	< 0.001*
TDI RV				
Free wall				
* S*′ (cm/s)	9.8 ± 2.0	3.7 [3.7–5.6]	− 4.9 ± 2.5	< 0.001*
Diastolic				
TV Doppler flow				
* E* (m/s)	0.9 ± 0.2	1.0 ± 0.2	0.06 ± 0.3	0.21
* A* (m/s)	0.8 ± 0.3	0.8 ± 0.2	− 0.1 ± 0.5	0.41
* E*/*A*	1.2 ± 0.5	1.1 [0.9–1.6]	0.2 ± 1.0	0.68
TDI RV				
Free wall				
* E*′ (cm/s)	16.8 ± 7.1	6.7 [5.0–8.0]	− 10.2 ± 6.4	< 0.001*
* A*′ (cm/s)	12.9 ± 4.2	5.4 ± 3.3	− 6.8 ± 4.7	< 0.001*
* E*/*E*′	6.1 ± 2.4	15.5 ± 5.1	9.8 ± 5.2	< 0.001*

*Means significant. *p *value determined with paired samples *t* test or Wilcoxon signed-rank test

*TAPSE* tricuspid annular plane systolic excursion, *TDI* tissue doppler image, *RV* right ventricle, *TV* tricuspid valve

### Relation to Clinical Parameters

There was a slight negative correlation between the age at surgery and the postoperative LV global peak strain (*R* = -0.41, *p* = 0.031).

There was no correlation of echocardiographic parameters and all other clinical parameters, such as postoperative RBBB or duration of pleural effusion.

### Intraobserver and Interobserver Variability

In general, there was a good to excellent inter and intraobserver agreement for the TDI and ST parameters (Table 5), with almost all the ICC > 0.9 in both intra and interobserver analysis.

**Table 5:** Intraclass correlation coefficient

	Intraobserver agreement		Interobserver agreement	
	ICC	95% CI	ICC	95% CI
TDI				
LV Lateral wall				
* S*′	0.987	0.973–0.994	0.855	0.690–0.932
* E*′	0.996	0.992–0.998	0.924	0.244–0.979
* A*′	1		0.162	
IVS				
* S*′	0.988	0.963–0.995	0.972	0.835–0.991
* E*′	0.996	0.992–0.998	0.919	0.412–0.976
* A*′	0.994	0.987–0.997	0.961	0.728–0.988
RV Lateral wall				
* S*′	0.994	0.988–0.997	0.987	0.850–0.996
* E*′	0.998	0.995–0.999	0.977	0.816–0.993
* A*′	0.782	0.584–0.895	-0.002	
Speckle tracking				
GLS	0.941	0.870–0.972	0.892	0.761–0.950
TGPS	0.969	0.935–0.985	0.961	0.917–0.981

*ICC* intraclass correlation coefficient*, CI* confidence interval, *TDI* tissue doppler mage, *LV* left ventricle, *IVS* interventricular septum *RV* right ventricle

## Discussion

In this study, systolic and diastolic function in ToF patients was evaluated by echocardiography before surgical repair and at discharge from the hospital after surgical correction. At hospital discharge, both LV and RV systolic and diastolic function were deteriorated, although this was more prominent for the RV. RV systolic and diastolic dysfunction was not related to postoperative RBBB or use of transannular patch. To our knowledge this is the first study that evaluates the ventricular function with new echocardiographic techniques before and shortly after surgery in a homogenous group of Fallot patients after corrective surgery.

### LV Function

In the present study, no change in FS was found in Fallot patients after surgical correction. LV FS has proven to be a quick, easy and reproducible tool to describe LV systolic performance and is still one of the methods used to asses LV performance [6, 21]. Its main disadvantage is the geometric assumption on which it is based, especially in CHD patients with very diverse ventricular shapes [22]. It has been shown previously that Tissue Doppler Imaging may unmask a decrease in LV systolic function, while FS still was in the normal range [15]. We found an increased *E*/*A* ratio, a decrease in global longitudinal strain and a decrease in systolic and diastolic tissue Doppler parameters of the interventricular septum. Even though the LV inflow parameters are age, heart rate and load dependent [23], TDI permits the direct quantification of myocardial velocities [12] with a relative geometric independence. Since TDI is based on Doppler it is angle-dependent and can therefore only be used in a longitudinal direction along the Doppler beam [6]. In comparison, ST is not angle-dependent, is not based on geometrical assumption and allows multidirectional assessment of ventricular function, making possible to study both regional and global function in both ventricles [13]. Although information about the immediate postoperative echocardiographic changes of LV function in Fallot patients in the literature is lacking, there is substantial evidence that even at an age of 5 years changes in LV function are present [8, 24]. In these studies, LV function was correlated to the amount of pulmonary regurgitation. In the present study we did not perform MRI, so exact quantification of pulmonary regurgitation was not possible. Furthermore, the RVH, immediately postoperatively with its restrictive physiology may in part prevent pulmonary regurgitation and may not be representative for the effects of volume overload by pulmonary regurgitation during long-term follow-up. LV function during follow-up has found to be deteriorated in many studies in TOF patients [25, 26]. The significance of diminished LV function has been underscored by its correlation to various clinical outcome parameters, including death, sustained ventricular tachycardia and diminished exercise tolerance [27, 28]. The underlying mechanism of diminished LV function is as yet incompletely understood [24]. Proposed mechanisms include ventricular–ventricular interactions and the insertion of a septal patch to close the ventricular septal defect. This is supported by the fact that only septal parameters (TDI and regional ST) in our study had significantly decreased after surgery. Moreover, Li et al. found that longitudinal septal peak strain was lower in patients with TOF than in control subjects, whereas LV lateral wall longitudinal strain did not differ between the groups [8]. Other possible mechanism are changes in aortic dynamics including decreased distensibility [29] which may be further aggravated by aortic root dilatation. If the immediate postoperative changes show a correlation to longer follow-up has still to be established.

### RV Function

In the present study RV systolic performance was decreased at discharge when studied with TAPSE. In addition, RV systolic and diastolic TDI parameters remained lower at discharge as well (*S*′, *E*′, *A*′ and *E*/*E*′). These findings are in line with previous studies investigating the early postoperative course in CHD patients [4, 15]. The importance of postoperative changes in RV function has previously been underscored by studying its relation to increased postoperative inotropic support [30]. In the present study, however, no correlation was found with the development of RBBB or persistent pleural drainage. Until now no clear explanation has been given for the early postoperative changes. Factors that have been mentioned include local tissue damage of the thin-walled anteriorly positioned RV, cardiopulmonary bypass, with less protection of the RV by cold cardioplegia, and pericardiotomy or pericardial adhesions [31]. In addition, the preoperative condition of Fallot patients is characterized by pressure and/or volume overload of the RV. During medium term follow-up RV deterioration in Fallot patients seems to persist [8, 26]. Furthermore, during long-term follow-up RV function continues to deteriorate. Contributing factors include pulmonary stenosis and/or insufficiency, and dyssynchronous contractions aggravated by QRS prolongation. This deterioration has also been associated with clinical factors including diminished exercise tolerance, ventricular arrhythmias and even cardiovascular death [27, 32, 33].

### Relation to Clinical Parameters

The best age for performing definitive corrective surgery for ToF is still a topic of discussion but there is a world-wide tendency to perform Fallot corrections as early as six months [34]. In a previous study by Li et al. [8] lower strain values of the RV were found in patients operated at an age above 12 months of age as compared to an age below 12 months. In the present study we found an effect of age at surgery on LV strain by the negative correlation between age at surgery and postoperative LV global peak strain. Although this can be interpreted as an advancement of later repair, this finding should be interpreted with caution because of the small number of patients and the rather weak correlation.

## Limitations

In the current study, only LV longitudinal strain analysis was performed. Image quality was insufficient to measure RV strain in a significant number of patients. However, the results of LV strain and of TDI RV data support and further extends previous data on short-term postoperative ventricular performance [19]. Since the focus of the present investigation was on short-term follow-up it was not as yet possible to correlate our findings to long-term complications. Future studies in which both postoperative echocardiography as well as long-term results are considered are necessary to solve this gap in knowledge. Finally, the small sample size of the present study precludes further subgroup analysis, e.g., with/without prior BT-shunt, the use of a transannular patch, or other confounding variables.

## Conclusion

There seems to be an impairment in left and right ventricle performance at discharge after Tetralogy of Fallot corrective surgery compared to preoperatively. This is better determined with Tissue Doppler imaging and Speckle tracking strain imaging than with conventional echocardiography. However, this is a pilot study with a small sample and a new study with larger population is needed to confirm our results.
